# Chromosomal radiosensitivity in head and neck cancer patients: evidence for genetic predisposition?

**DOI:** 10.1038/sj.bjc.6604345

**Published:** 2008-04-15

**Authors:** K De Ruyck, V de Gelder, M Van Eijkeren, T Boterberg, W De Neve, A Vral, H Thierens

**Affiliations:** 1 Department of Human Anatomy, Embryology, Histology and Medical Physics, Ghent University, Proeftuinstraat 86, Ghent B-9000, Belgium; 2 Department of Radiation Oncology, Ghent University Hospital, De Pintelaan 185, Ghent B-9000, Belgium

**Keywords:** head and neck cancer, chromosomal radiosensitivity, genetic predisposition

## Abstract

The association between chromosomal radiosensitivity and genetic predisposition to head and neck cancer was investigated in this study. In all, 101 head and neck cancer patients and 75 healthy control individuals were included in the study. The G_2_ assay was used to measure chromosomal radiosensitivity. The results demonstrated that head and neck cancer patients had a statistically higher number of radiation-induced chromatid breaks than controls, with mean values of 1.23 and 1.10 breaks per cell, respectively (*P*<0.001). Using the 90th percentile of the G_2_ scores of the healthy individuals as a cutoff value for chromosomal radiosensitivity, 26% of the cancer patients were radiosensitive compared with 9% of the healthy controls (*P*=0.008). The mean number of radiation-induced chromatid breaks and the proportion of radiosensitive individuals were highest for oral cavity cancer patients (1.26 breaks per cell, 38%) and pharynx cancer patients (1.27 breaks per cell, 35%). The difference between patients and controls was most pronounced in the lower age group (⩽50 years, 1.32 breaks per cell, 38%) and in the non- and light smoking patient group (⩽10 pack-years, 1.28 breaks per cell, 46%). In conclusion, enhanced chromosomal radiosensitivity is a marker of genetic predisposition to head and neck cancer, and the genetic contribution is highest for oral cavity and pharynx cancer patients and for early onset and non- and light smoking patients.

Enhanced chromosomal radiosensitivity has been observed in a large number of patients with cancer-prone genetic diseases such as ataxia–telangiectasia and Nijmegen breakage syndrome (Gatti, 2001). Several studies have shown that enhanced chromosomal radiosensitivity is also present in a significant proportion of cancer patients (Scott *et al*, 1994, 1998, 1999; Parshad *et al*, 1996; Patel *et al*, 1997; Terzoudi *et al*, 2000; Baria *et al*, 2001, 2002; Buchholz and Wu, 2001; Papworth *et al*, 2001; Riches *et al*, 2001; Baeyens *et al*, 2002, 2005; Ban *et al*, 2004; Howe *et al*, 2005; Kolusayin Ozar and Orta, 2005; Mozdarani *et al*, 2005; Distel *et al*, 2006; Lisowska *et al*, 2006; Varga *et al*, 2006). The majority of data have been collected in studies considering breast cancer patients whose lymphocytes were irradiated in the G_2_ phase of the cell cycle (Scott *et al*, 1994, 1999; Parshad *et al*, 1996; Patel *et al*, 1997; Terzoudi *et al*, 2000; Baria *et al*, 2001; Buchholz and Wu, 2001; Riches *et al*, 2001; Baeyens *et al*, 2002, 2005; Howe *et al*, 2005; Djuzenova *et al*, 2006; Docherty *et al*, 2007). In a high number of these studies, it was found that approximately 40% of breast cancer patients show an increased G_2_ chromosomal radiosensitivity (Scott *et al*, 1994, 1999; Baria *et al*, 2001; Buchholz and Wu, 2001; Riches *et al*, 2001; Baeyens *et al*, 2002, 2005). As it was also shown that healthy first-degree relatives of breast cancer patients have an enhanced G_2_ sensitivity (Parshad *et al*, 1996; Patel *et al*, 1997), it has been suggested that this elevated *in vitro* radiosensitivity may be a marker of low-penetrant cancer-predisposing genes (Roberts *et al*, 1999).

There is also indirect evidence for the existence of low-penetrant predisposing genetic factors for head and neck cancers (Sturgis *et al*, 2004). Although tobacco and alcohol consumption play a major role in the aetiology of head and neck squamous cell carcinoma (HNSCC), several large family studies demonstrated a three- to eightfold increased risk of HNSCC in first-degree relatives of patients with HNSCC (Copper *et al*, 1995; Foulkes *et al*, 1995, 1996). Furthermore, only a fraction of frequently exposed individuals develop HNSCC and up to 20% of all HNSCC patients develop the disease without any exposure to the main risk factors (Sturgis *et al*, 2004). Finally, data emerging from case–control studies using several phenotypic and genotypic assays support the hypothesis of genetic susceptibility for HNSCC (Sturgis and Wei, 2002). Until recently, most of the studies evaluating phenotypic assays as possible marker for head and neck cancer susceptibility focused on the analysis of chromosomal damage in lymphocytes after mutagen exposure (Schantz and Hsu, 1989; Schantz *et al*, 1989; Bondy *et al*, 1993; Spitz *et al*, 1993; Cloos *et al*, 1996; Wang *et al*, 1998; Wu *et al*, 1998; Zhu *et al*, 2002; Szekely *et al*, 2005; Xiong *et al*, 2007). Studies analysing chromosomal damage after *in vitro* irradiation are, however, limited and often comprise a low number of patients (Terzoudi *et al*, 2000; Papworth *et al*, 2001; Ban *et al*, 2004; Distel *et al*, 2006; Lisowska *et al*, 2006). Nevertheless, both Terzoudi *et al* (2000) and Lisowska *et al* (2006) reported enhanced chromosomal radiosensitivity in larynx cancer patients, and Papworth *et al* (2001) observed increased chromosomal radiosensitivity in young head and neck cancer patients.

In the present study, we investigated the association between chromosomal radiosensitivity and genetic predisposition to head and neck cancer using the G_2_ assay for peripheral blood lymphocytes. An analysis of the data according to tumour histology and tumour location was performed. In addition, the influence of age of onset of the disease and the effect of smoking habits on genetic predisposition was investigated.

## MATERIALS AND METHODS

### Population

The study population consisted of 101 head and neck cancer patients recruited from the Radiation Oncology Department of the Ghent University Hospital between January 2003 and October 2005. All patients were newly diagnosed, previously untreated and histologically confirmed. The cases included 83 squamous cell carcinomas (SCCs), 12 adenocarcinomas (ACs) and 6 other or unknown carcinomas. A total of 13 patients had cancer of the oral cavity, 37 patients suffered from pharyngeal cancer, 29 patients had laryngeal cancer and 22 patients were diagnosed with sinus cancer or cancer of unknown primary. The population consisted of 84 men and 17 women, and the mean age of the patients at the time of treatment was 60 years (range: 33–91 years) (Table 1).

From each patient in the study, a heparinised blood sample was taken before therapy and the G_2_ assay was performed on the sampling day. At the same time, parallel cultures from healthy individuals were started. The samples of the healthy control individuals were collected at the Occupational Medicine Department of the Ghent University Hospital. Details of the control population are described in Table 1. The study was approved by the Ethical Committee of the Ghent University Hospital. All study participants signed an informed consent form and completed a detailed questionnaire on smoking and drinking habits. Smoking was quantified by the degree of tobacco consumption categorised as pack-years (1 pack-year=20 cigarettes per day for 1 year). Alcohol exposure was determined by the number of alcoholic drinks per week. For four patients and two control individuals, no smoking and drinking data were available.

### G_2_ assay

The G_2_ assay procedure of the Paterson Institute (Manchester) was applied with some minor changes. Briefly, heparinised blood was kept at room temperature before culturing within 4 h after prelevation. To a tissue culture flask (25 cm^2^), 0.5 ml blood was added to 4.5 ml complete RPMI-1640 culture medium supplemented with 10% fetal calf serum (Invitrogen, Merelbeke, Belgium), 1% L-glutamine, 50 U ml^−1^ penicillin and 50 μg ml^−1^ streptomycin. The lymphocytes were stimulated to divide with 1% phytohemagglutinin (Invitrogen). Per donor two cultures were started, one served as control, the other for *in vitro* irradiation. After 70 h incubation in a CO_2_ incubator at 37°C, the cultures were irradiated with a dose of 0.4 Gy ^60^Co rays at 37°C. At 30 min post irradiation, 75 μl colcemid (final concentration 0.15 μg ml^−1^; Sigma-Aldrich, Bornem, Belgium) was added to block the cells at metaphase, and at 90 min post irradiation the cultures were arrested by putting them on ice for 5 min. The cells were harvested by centrifugation of the samples, and the cell pellets were resuspended in 5 ml of 0.075 M KCl for 15 min on ice. After the hypotonic shock, the cells were fixed three times in 5 ml cold methanol/acetic acid (3 : 1). Finally, cells were dropped on clean slides and stained with 4% Azur B SCN solution (Polylab, Merksem, Belgium). Fifty well-spread metaphases were analysed by two independent scorers on coded slides for the appearance of chromatid breaks. All types of single chromatid breaks where a clear discontinuity was present were scored (Bryant *et al*, 2002).

### Statistical analysis

Statistical analysis was performed by SPSS 12.0 software (SPSS Inc., Chicago, IL, USA). For the comparison of the mean G_2_ scores between different groups, the Mann–Whitney test was applied. Differences in the proportions of radiosensitive individuals in the different populations were compared using the *χ*^2^ test. Unconditional logistic regression analyses were performed to calculate ORs (odds ratios) and 95% CIs (95% confidence intervals). Pearson correlation coefficients were used to study the correlation between aberration frequencies, age, smoking and drinking.

## RESULTS

Chromosomal radiosensitivity G_2_ data were collected for 75 healthy individuals and 101 head and neck cancer patients. For each sample, the spontaneous yield of chromatid breaks was subtracted from the yield in irradiated cells to obtain the radiation-induced aberration yield.

**Figure 1:**
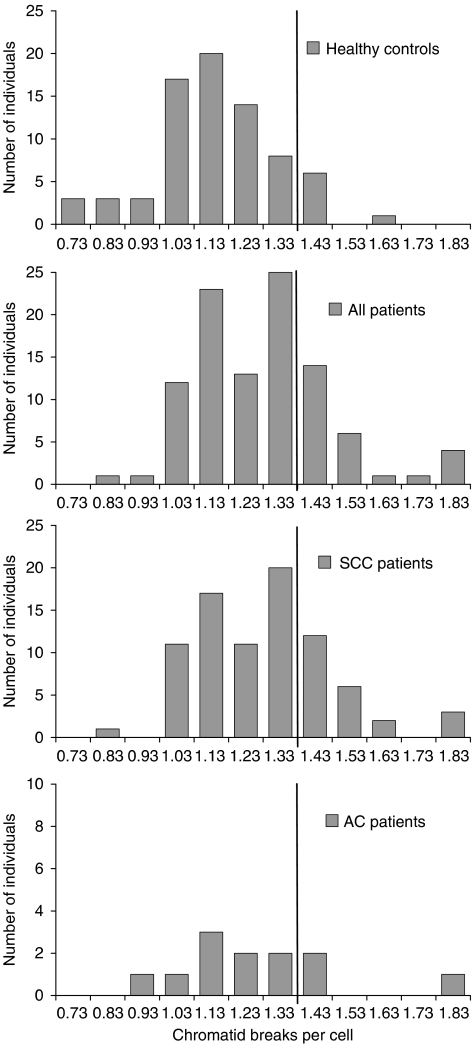
Distribution of radiation-induced G_2_ chromatid breaks in healthy individuals (*n*=75), all head and neck cancer (*n*=101), SCC (*n*=83) and AC patients (*n*=12). The vertical line represents the 90th percentile cutoff point.

**Figure 2:**
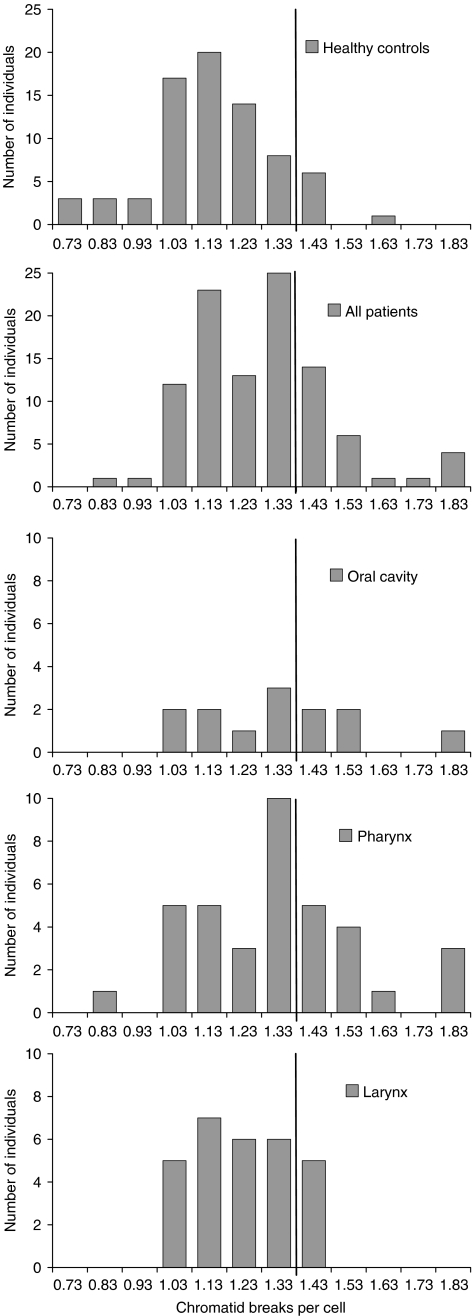
Distribution of radiation-induced G_2_ chromatid breaks in healthy individuals (*n*=75), all head and neck cancer (*n*=101), oral cavity cancer (*n*=13), pharynx cancer (*n*=37) and larynx cancer patients (*n*=29). The vertical line represents the 90th percentile cutoff point.

The results for the head and neck cancer patients and for the healthy individuals are summarised in Table 2 and graphically displayed in Figures 1 and 2. The average yield of radiation-induced chromatid breaks per cell was 1.10 for healthy individuals and 1.23 for all head and neck cancer patients (*P*<0.001). According to tumour histology, patients with SCC and AC were considered. Patients with SCC had 1.23 breaks per cell (*P*<0.001), whereas those with AC had 1.21 breaks per cell (*P*=0.166). Using the 90th percentile of the G_2_ scores of the healthy individuals as a cutoff value for chromosomal radiosensitivity (1.33 breaks per cell), 26% of all cancer patients, 28% of SCC patients and 25% of AC patients were radiosensitive compared with 9% of the healthy individuals (*P*=0.008, 0.005 and 0.261, respectively). This corresponds, respectively, to ORs with respect to cancer incidence of 3.37 (*P*=0.008), 3.72 (*P*=0.005) and 3.24 (*P*=0.130) for individuals with G_2_ scores exceeding 1.33 breaks per cell (Table 2). According to anatomic tumour location, three patients subgroups were considered: (1) patients with oral cavity cancer, (2) patients with pharyngeal cancer and (3) patients with laryngeal cancer. Patients from each subgroup had significantly more chromatid breaks per cell compared with the individuals from the healthy control group (*P*=0.021, <0.001 and 0.024, respectively). On the basis of the 90th percentile cutoff value, 38% of the oral cavity cancer patients, 35% of the pharynx cancer patients and 17% of the larynx cancer patients were radiosensitive (*P*=0.018, 0.002 and 0.419, respectively). Consequently, individuals with increased chromosomal radiosensitivity had a six-, five- or two-fold increased risk to develop oral cavity cancer, pharynx cancer or larynx cancer (*P*=0.009, 0.002 or 0.265), respectively.

For further analyses, the patients were stratified according to age, smoking habits and alcohol intake, and chromosomal radiosensitivity was evaluated in the subsequent subgroups. The results of these analyses are presented in Table 3. No significant effect of age was seen on radiation-induced aberrations in individual patients and controls (*P*>0.100), but stratified analysis showed the highest mean G_2_ scores for the youngest patients. Patients with ages ⩽50, between >50 and ⩽70, and >70 years had 1.32, 1.22 and 1.18 breaks per cell, respectively. Except for the oldest patient group (*P*=0.269), these results were significantly different from the control population (*P*=0.004 and <0.001, respectively). Using the cutoff value of the healthy control group, 38% of the patients with age ⩽50 years (*P*=0.009) were radiosensitive. Consequently, radiosensitive individuals ⩽50 years had a six-fold increased risk for developing head and neck cancer (*P*=0.007). There was also no correlation between smoking and radiation-induced aberrations in patients and controls (*P*>0.130). Nevertheless, the highest mean G_2_ scores were found for non- and light smoking patients. Patients with ⩽10, between >10 and ⩽25, and >25 pack-years had 1.28, 1.22 and 1.21 breaks per cell, respectively. The results of all subgroups defined according to smoking habits were statistically significant compared with the control population (*P*=0.006, 0.007 and 0.001, respectively). On the basis of the cutoff value of the healthy control group, 46% of the patients with ⩽10 pack-years (*P*=0.003) were radiosensitive. Accordingly, an OR of 8.33 (*P*=0.002) was found for non- and light smoking individuals. A similar trend was not seen for alcohol intake in the patient population, nor for any of the three parameters in the control population.

## DISCUSSION

The association between G_2_ chromosomal radiosensitivity and genetic predisposition to head and neck cancer was investigated in the present study. Moreover, an analysis of the data according to tumour histology and tumour location was performed, and the influence of age of onset of the disease and the effect of smoking habits on genetic predisposition were investigated.

Analysis of the data shows that head and neck cancer patients have a significantly higher mean number of *in vitro* radiation-induced chromatid breaks per cell than healthy individuals (1.23 *vs* 1.10 breaks per cell, *P*<0.001). The observed elevated chromosomal radiosensitivity was independent of tumour histology and tumour location. Patients with SCC and AC, as well as patients with oral cavity, pharynx and larynx cancers, all showed an increased mean number of radiation-induced chromosomal aberrations compared with the control group. Except for the AC patients, all results were statistically significant. With respect to laryngeal cancer, these results are in agreement with the findings of Terzoudi *et al* (2000) and Lisowska *et al* (2006), who both reported enhanced chromosomal radiosensitivity in larynx cancer patients. This increase was statistically significant in the latter study, but in the study of Terzoudi *et al* (2000), the statistical significance was not mentioned. In the study of Lisowska *et al*, a very different experimental protocol was used compared with the one applied in the present study. Although five times higher irradiation doses were used (2 *vs* 0.4 Gy in the present study), five times lower mean G_2_ values were obtained for larynx cancer patients (0.22 *vs* 1.18 breaks per cells in the present study). This discrepancy is at least partly due to the fact that in the study of Lisowska *et al*, chromatid breaks were analysed in metaphase cells 4.5 h post irradiation, whereas in the present study chromatid breaks were analysed in metaphase cells 1.5 h post irradiation. An exponential decline of chromatid breaks with time after irradiation has been demonstrated in the past by our research group and supports the observed differences in absolute chromatid break numbers in both studies (Vral *et al*, 2002). Concerning various head and neck cancer patients, Papworth *et al* (2001) reported a nonsignificant increase in mean radiation-induced chromosomal aberrations in these patients. Next to the positive association found between high levels of radiation-induced chromatid breaks and increased risk for head and neck cancer, several studies also reported a positive association between high frequencies of chromatid breaks after bleomycin or benzo[*a*]pyrene diol epoxide (BPDE) exposure and elevated risk for head and neck cancer (Cloos *et al*, 1996; Wang *et al*, 1998; Wu *et al*, 1998; Zhu *et al*, 2002; Szekely *et al*, 2005; Xiong *et al*, 2007). As radiation as well as bleomycin and BPDE cause DNA damage, both assays are linked to DNA repair capacity. Consequently, the observed enhanced chromosomal radiosensitivity in cancer patients indicates that suboptimal repair of DNA damage contributes to the genetic susceptibility to head and neck cancer. Alternatively, the observed enhanced chromosomal radiosensitivity may also result from a less efficient G_2_/M checkpoint, as it has been shown that G_2_ checkpoint abrogation comprises repair of chromosome damage and that defects in the G_2_/M checkpoint are associated with increased cancer risk (Terzoudi *et al*, 2005; Wu *et al*, 2005; Zheng *et al*, 2005).

Although in this study the mean number of radiation-induced aberrations was significantly higher in the studied cancer population compared with the healthy control population, an overlap exists between both groups. Therefore, a cutoff value, above which an individual can be considered as radiosensitive, was determined (Scott *et al*, 1999). With this value, the proportion of radiosensitive individuals can be compared between different populations. As in most studies, the 90th percentile of the normal population was considered as cutoff point. Using this threshold for chromosomal radiosensitivity, 26% of the head and neck cancer patients were radiosensitive compared with 9% of the healthy individuals (*P*=0.008). A high proportion of radiosensitive cases (31%) among various head and neck cancer patients have also been reported by Papworth *et al* (2001). Subdividing our large patient group according to anatomic location of the tumour revealed that the high G_2_ scores were mainly attributable to the oral cavity and pharyngeal cancer patients of which 38 and 35%, respectively, were radiosensitive (*P*=0.018 and 0.002). Of the laryngeal cancer patients, in contrast, only 17% of the patients were radiosensitive (*P*=0.419). In the study of Lisowska *et al* (2006), a higher and significant proportion of radiosensitive individuals (40%) among larynx cancer patients was reported. Because a very different experimental protocol is used by Lisowska *et al*, compared with the present study, this could explain the difference in the number of radiosensitive larynx cancer patients.

In this study, no correlation has been found between radiation-induced aberration frequency and age, pack-years of smoking and drinks per week in patients and controls. Concerning age, this has also been published in other studies (Scott *et al*, 1999; Riches *et al*, 2001; Vral *et al*, 2007). For smoking and drinking exposure, no data are available in the literature for their effect on the level of radiation-induced aberrations. It has, however, been shown that age, smoking and alcohol intake do not influence mutagen-induced chromosomal aberrations (Cloos *et al*, 1996). Despite the lack of correlation on individual level, grouped analysis showed that patients ⩽50 years and with ⩽10 pack-years had the highest mean number of chromatid breaks. On the basis of the 90th percentile cutoff value, 38% of the patients aged 50 years or less were radiosensitive (*P*=0.009). Similar proportions of *in vitro* radiosensitive young head and neck cancer patients have also been reported by Papworth *et al* (2001) (38%, ⩽45 years). Our patient group included only five patients ⩽45 years but 60% of them were *in vitro* radiosensitive (*P*=0.008). These results indicate that the genetic contribution, as measured by *in vitro* radiosensitivity, to head and neck cancer risk is higher for early onset cases. This finding for head and neck cancer patients differs from that for breast cancer patients in that no age dependence for G_2_ sensitivity could be found in the latter group (Baeyens *et al*, 2005). As cancer at young age is a hallmark of heredity, this indicates that individuals at risk for early onset head and neck cancer possess a higher number of low-penetrant predisposing genes or a number of highly penetrant predisposing genes involved in DNA repair pathways compared with late-onset head and neck cancer patients and early onset breast cancer patients. Concerning tobacco exposure, it was found that non- and light smoking patients had the highest mean number of radiation-induced chromatid breaks. This indicates the existence of a larger genetic predisposition in non- and moderately tobacco-exposed individuals and supports the possibility of important gene–environment interactions in the development of head and neck cancer (Xiong *et al*, 2007).

In conclusion, this study supports the fact that chromosomal radiosensitivity of lymphocytes is a marker of genetic predisposition to head and neck cancer. Analyses of different subgroups revealed that the genetic contribution is highest for oral cavity and pharynx cancer patients and is more pronounced for early onset and non- and light smoking patients.

## Figures and Tables

**Table 1:** Characteristics of the study population

	**Controls (%)**	**Patients (%)**
**Characteristic**	**(*n*=75)**	**(*n*=101)**
*Sex*		
Male	41 (54.7)	84 (83.2)
Female	34 (45.3)	17 (16.8)
Mean age ±s.d.	45±13.7	60±10.0
		
*Anatomic tumour location*		
Oral cavity	—	13 (12.9)
Pharynx	—	37 (36.6)
Larynx	—	29 (28.7)
Other and unknown	—	22 (21.8)
		
*Tumour histology*		
Squamous cell carcinoma (SCC)	—	83 (82.2)
Adenocarcinoma	—	12 (11.9)
Other and unknown	—	6 (5.9)
Mean pack-years ±s.d.	7±11.6	33±21.5
Mean drinks per week ±s.d.	6±5.4	28±27.9

**Table 2:** Overview of the G_2_ data

		**Head and neck cancer patients**					
			**Tumour histology**		**Tumour location**		
	**Controls**	**All**	**SCC**	**AC**	**Oral cavity**	**Pharynx**	**Larynx**
Population size	75	101	83	12	13	37	29
Mean±s.d.^a^	1.10±0.18	1.23±0.20	1.23±0.20	1.21±0.24	1.26±0.23	1.27±0.24	1.18±0.14
Range^a^	0.64–1.54	0.75–1.81	0.75–1.81	0.90–1.76	0.94–1.76	0.75–1.81	0.96–1.43
*P*-value^b^		<0.001	<0.001	0.166	0.021	<0.001	0.024
Cut-off value^c^	1.33						
% >cut off value	9	26	28	25	38	35	17
*P*-value^d^		0.008	0.005	0.261	0.018	0.002	0.419
OR (95% CI)	Ref^e^	3.37 (1.37–8.26)	3.72 (1.49–9.29)	3.24 (0.71–14.82)	6.07 (1.56–23.69)	5.26 (1.88–14.74)	2.02 (0.59–6.98)
*P*-value^f^		0.008	0.005	0.130	0.009	0.002	0.265

AD, adenocarcinoma; CI, confidence interval; OR, odds ratio; SCC, squamous cell carcinoma.

a Number of chromatid breaks per cell.

b Mann–Whitney test.

c 90th percentile of the control population.

d *χ*^2^ test.

e Ref, reference: control individuals with ⩽1.33 breaks per cell were used as reference.

f Logistic regression.

**Table 3:** Stratification analysis of G_2_ scores in head and neck cancer patients according to age and smoking

**Subgroup**	**Population size**	**Mean G_2_ score^a^**	***P*-value^b^**	**% >cutoff value^c^**	***P*-value^d^**	**OR (95% CI)**	***P*-value^e^**
*Age (years)*							
⩽50	16	1.32	0.004	38	0.009	5.83 (1.63–20.89)	0.007
>50 to ⩽70	71	1.22	<0.001	27	0.009	3.55 (1.39–9.08)	0.008
>70	14	1.18	0.269	7	0.788	0.75 (0.09–6.60)	0.793
							
*Smoking (PY)*							
⩽10	13	1.28	0.006	46	0.003	8.33 (2.18–31.78)	0.002
>10 to ⩽25	25	1.22	0.007	24	0.113	3.07 (0.92–10.22)	0.068
>25	59	1.21	0.001	20	0.120	2.48 (0.91–6.77)	0.076

CI, confidence interval; OR, odds ratio; PY, pack-years.

a Number of chromatid breaks per cell.

b Mann–Whitney test, compared with the control population.

c Cutoff value=90th percentile of the control population=1.33 breaks per cell.

d *χ*^2^ test, compared with the proportion of control individuals with a G_2_ score > cutoff value.

e Logistic regression, control individuals with ⩽1.33 breaks per cell were used as reference.

## References

[bib1] Baeyens A, Thierens H, Claes K, Poppe B, Messiaen L, De Ridder L, Vral A (2002) Chromosomal radiosensitivity in breast cancer patients with a known or putative genetic predisposition. Br J Cancer 87: 1379–1385 12454765 10.1038/sj.bjc.6600628 PMC2376291

[bib2] Baeyens A, Van Den Broecke R, Makar A, Thierens H, De Ridder L, Vral A (2005) Chromosomal radiosensitivity in breast cancer patients: influence of age of onset of the disease. Oncol Rep 13: 347–353 15643523

[bib3] Ban S, Konomi C, Iwakawa M, Yamada S, Ohno T, Tsuji H, Noda S, Matui Y, Harada Y, Cologne JB, Imai T (2004) Radiosensitivity of peripheral blood lymphocytes obtained from patients with cancers of the breast, head and neck or cervix as determined with a micronucleus assay. J Radiat Res 45: 535–541 15635263 10.1269/jrr.45.535

[bib4] Baria K, Warren C, Eden OB, Roberts SA, West CM, Scott D (2002) Chromosomal radiosensitivity in young cancer patients: possible evidence of genetic predisposition. Int J Radiat Biol 78: 341–346 12020425 10.1080/09553000110117359

[bib5] Baria K, Warren C, Roberts SA, West CM, Scott D (2001) Chromosomal radiosensitivity as a marker of predisposition to common cancers? Br J Cancer 84: 892–896 11286467 10.1054/bjoc.2000.1701 PMC2363837

[bib6] Bondy ML, Spitz MR, Halabi S, Fueger JJ, Schantz SP, Sample D, Hsu TC (1993) Association between family history of cancer and mutagen sensitivity in upper aerodigestive tract cancer patients. Cancer Epidemiol Biomarkers Prev 2: 103–106 7682127

[bib7] Bryant PE, Gray L, Riches AC, Steel CM, Finnon P, Howe O, Kesterton I, Vral A, Curwen GB, Smart V, Tawn EJ, Whitehouse CA (2002) The G_2_ chromosomal radiosensitivity assay. Int J Radiat Biol 78: 863–866 12428927 10.1080/09553000210144484

[bib8] Buchholz TA, Wu XF (2001) Radiation-induced chromatid breaks as a predictor of breast cancer risk. Int J Radiat Oncol Biol Phys 49: 533–537 11173151 10.1016/s0360-3016(00)01502-9

[bib9] Cloos J, Spitz MR, Schantz SP, Hsu TC, Zhang ZF, Tobi H, Braakhuis BJM, Snow GB (1996) Genetic susceptibility to head and neck squamous cell carcinoma. J Natl Cancer Inst 88: 530–535 8606381 10.1093/jnci/88.8.530

[bib10] Copper MP, Jovanovic A, Nauta JJP, Braakhuis BJM, De Vries N, Van der Waal I, Snow GB (1995) Role of genetic factors in the etiology of squamous cell carcinoma of the head and neck. Arch Otolaryngol Head Neck Surg 121: 157–160 7840922 10.1001/archotol.1995.01890020019005

[bib11] Distel LVR, Neubauer S, Keller U, Sprung CN, Sauer R, Grabenbauer GG (2006) Individual differences in chromosomal aberrations after *in vitro* irradiation of cells from healthy individuals, cancer and cancer susceptibility syndrome patients. Radiother Oncol 81: 257–263 17113667 10.1016/j.radonc.2006.10.012

[bib12] Djuzenova CS, Muhl B, Fehn M, Oppitz U, Muller B, Flentje M (2006) Radiosensitivity in breast cancer assessed by the Comet and micronucleus assays. Br J Cancer 94: 1194–1203 16538220 10.1038/sj.bjc.6603005 PMC2361251

[bib13] Docherty Z, Georgiou A, Langman C, Kesterton I, Rose S, Camplejohn R, Ball J, Barwell J, Gilchrist R, Pangon L, Berg J, Hodgson S (2007) Is chromosome radiosensitivity and apoptotic response to irradiation correlated with cancer susceptibility? Int J Radiat Biol 83: 1–12 17357435 10.1080/09553000600932968

[bib14] Foulkes WD, Brunet JS, Kowalski LP, Narod SA, Franco EL (1995) Family history of cancer is a risk factor for squamous cell carcinoma of the head and neck in Brazil: a case–control study. Int J Cancer 63: 769–773 8847131 10.1002/ijc.2910630603

[bib15] Foulkes WD, Brunet JS, Sieh W, Black MJ, Shenouda G, Narod SA (1996) Familial risks of squamous cell carcinoma of the head and neck: retrospective case–control study. BMJ 313: 716–721 8819440 10.1136/bmj.313.7059.716 PMC2352110

[bib16] Gatti RA (2001) The inherited basis of human radiosensitivity. Acta Oncol 40: 702–711 11765064 10.1080/02841860152619115

[bib17] Howe OL, Daly PA, Seymour C, Ormiston W, Nolan C, Mothersill C (2005) Elevated G_2_ chromosomal radiosensitivity in Irish breast cancer patients: a comparison with other studies. Int J Radiat Biol 81: 373–378 16076752 10.1080/09553000500147642

[bib18] Kolusayin Ozar MO, Orta T (2005) The use of chromosome aberrations in predicting breast cancer risk. J Exp Clin Cancer Res 24: 217–222 16110754

[bib19] Lisowska H, Lankoff A, Wieczorek A, Florek A, Kuszewski T, Gozdz S, Wojcik A (2006) Enhanced chromosomal radiosensitivity in peripheral blood lymphocytes of larynx cancer patients. Int J Radiat Oncol Biol Phys 6: 1245–1252 10.1016/j.ijrobp.2006.07.1370 17145539

[bib20] Mozdarani H, Mansouri Z, Haeri SA (2005) Cytogenetic radiosensitivity of G(0)-lymphocytes of breast and esophageal cancer patients as determined by micronucleus assay. J Radiat Res 46: 111–116 15802866 10.1269/jrr.46.111

[bib21] Papworth R, Slevin N, Roberts SA, Scott D (2001) Sensitivity to radiation-induced chromosome damage may be a marker of genetic predisposition in young head and neck cancer patients. Br J Cancer 84: 776–782 11259091 10.1054/bjoc.2000.1692 PMC2363817

[bib22] Parshad R, Price FM, Bohr VA, Cowans KH, Zujewski JA, Sanford KK (1996) Deficient DNA repair capacity, a predisposing factor in breast cancer. Br J Cancer 74: 1–5 8679441 10.1038/bjc.1996.307 PMC2074608

[bib23] Patel RK, Trivedi AH, Arora DC, Bhatavdekar JM, Patel DD (1997) DNA repair proficiency in breast cancer patients and their first-degree relatives. Int J Cancer 73: 20–24 9334804 10.1002/(sici)1097-0215(19970926)73:1<20::aid-ijc4>3.0.co;2-3

[bib24] Riches AC, Bryant PE, Steel CM, Gleig A, Robertson AJ, Preece PE, Thompson AM (2001) Chromosomal radiosensitivity in G_2_-phase lymphocytes identifies breast cancer patients with distinctive tumour characteristics. Br J Cancer 85: 1157–1161 11710829 10.1054/bjoc.2001.2086 PMC2375149

[bib25] Roberts SA, Spreadborough AR, Bulman B, Barber JBP, Evans DGR, Scott D (1999) Heritability of cellular radiosensitivity: a marker of low-penetrance predisposition genes in breast cancer. Am J Human Genet 65: 784–794 10441587 10.1086/302544 PMC1377987

[bib26] Schantz SP, Hsu TC (1989) Mutagen-induced chromosome fragility within peripheral blood lymphocytes of head and neck cancer patients. Head Neck 11: 337–342 2473966 10.1002/hed.2880110409

[bib27] Schantz SP, Hsu TC, Ainslie N, Moser RP (1989) Young adults with head and neck cancer express increased susceptibility to mutagen-induced chromosome damage. JAMA 262: 3313–3315 2479781

[bib28] Scott D, Barber JBP, Levine EL, Burrill W, Roberts SA (1998) Radiation-induced micronucleus induction in lymphocytes identifies a high frequency of radiosensitive cases among breast cancer patients: a test for predisposition? Br J Cancer 77: 614–620 9484819 10.1038/bjc.1998.98 PMC2149942

[bib29] Scott D, Barber JBP, Spreadborough AR, Burrill W, Roberts SA (1999) Increased chromosomal radiosensitivity in breast cancer patients: a comparison of two assays. Int J Radiat Biol 75: 1–10 9972785 10.1080/095530099140744

[bib30] Scott D, Spreadborough A, Levine E, Roberts SA (1994) Genetic predisposition in breast cancer. Lancet 344: 444 7968110 10.1016/s0140-6736(94)90615-7

[bib31] Spitz MR, Fueger JJ, Halabi S, Schantz SP, Sample D, Hsu TC (1993) Mutagen sensitivity in upper aerodigestive tract cancer: a case-control analysis. Cancer Epidemiol Biomarkers Prev 2: 329–333 7688625

[bib32] Sturgis EM, Wei Q, Spitz MR (2004) Descriptive epidemiology and risk factors for head and neck cancer. Semin Oncol 31: 726–733 15599850 10.1053/j.seminoncol.2004.09.013

[bib33] Sturgis EM, Wei QY (2002) Genetic susceptibility – molecular epidemiology of head and neck cancer. Curr Opin Oncol 14: 310–317 11981277 10.1097/00001622-200205000-00010

[bib34] Szekely G, Remenar E, Kasler M, Gundy S (2005) Mutagen sensitivity of patients with cancer at different sites of the head and neck. Mutagenesis 20: 381–385 16105906 10.1093/mutage/gei051

[bib35] Terzoudi GI, Jung T, Hain J, Vrouvas J, Margaritis K, Donta-Bakoyianni C, Makropoulos V, Angelakis P, Pantelias GE (2000) Increased G_2_ chromosomal radiosensitivity in cancer patients: the role of cdk1/cyclin-B activity level in the mechanisms involved. Int J Radiat Biol 76: 607–615 10866282 10.1080/095530000138268

[bib36] Terzoudi GI, Manola KN, Pantelias GE, Iliakis G (2005) Checkpoint abrogation in G(2) compromises repair of chromosomal breaks in ataxia telangiectasia cells. Cancer Res 65: 11292–11296 16357135 10.1158/0008-5472.CAN-05-2148

[bib37] Varga D, Hoegel J, Maier C, Jainta S, Hoehne M, Patino-Garcia B, Michel I, Schwarz-Boeger U, Kiechle M, Kreienberg R, Vogel W (2006) On the difference of micronucleus frequencies in peripheral blood lymphocytes between breast cancer patients and controls. Mutagenesis 21: 313–320 16928695 10.1093/mutage/gel035

[bib38] Vral A, Thierens H, Baeyens A, De Ridder L (2002) Induction and disappearance of G_2_ chromatid breaks in lymphocytes after low doses of low-LET gamma-rays and high-LET fast neutrons. Int J Radiat Biol 78: 249–257 12020436 10.1080/09553000110102003

[bib39] Vral A, Thierens H, Baeyens A, De Ridder L (2007) Chromosomal aberrations and *in vitro* radiosensitivity: intra-individual *versus* inter-individual variability. Toxicol Lett 149: 345–352 10.1016/j.toxlet.2003.12.044 15093280

[bib40] Wang LE, Sturgis EM, Eicher SA, Spitz MR, Hong WK, Wei QY (1998) Mutagen sensitivity to benzo(*a*)pyrene diol epoxide and the risk of squamous cell carcinoma of the head and neck. Clin Cancer Res 4: 1773–1778 9676854

[bib41] Wu XF, Gu J, Hong WK, Lee JJ, Amos CI, Jiang H, Winn RJ, Fu KKW, Cooper J, Spitz MR (1998) Benzo[*a*]pyrene diol epoxide and bleomycin sensitivity and susceptibility to cancer of upper aerodigestive tract. J Natl Cancer Inst 90: 1393–1399 9747870 10.1093/jnci/90.18.1393

[bib42] Wu XF, Roth JA, Zhao H, Luo S, Zheng YL, Chiang S, Spitz MR (2005) Cell cycle checkpoints, DNA damage/repair, and lung cancer risk. Cancer Res 65: 349–357 15665313

[bib43] Xiong P, Hu Z, Li C, Wang LE, El-Naggar AK, Sturgis EM, Wei Q (2007) *In vitro* benzo[*a*]pyrene diol epoxide-induced DNA damage and chromosomal aberrations in primary lymphocytes, smoking, and risk of squamous cell carcinoma of the head and neck. Int J Cancer 121: 2735–2740 17724733 10.1002/ijc.23054

[bib44] Zheng YL, Loffredo CA, Alberg AJ, Yu ZP, Jones RT, Perlmutter D, Enewold L, Krasna MJ, Yung R, Shields PG, Harris CC (2005) Less efficient G(2)–M checkpoint is associated with an increased risk of lung cancer in African Americans. Cancer Res 65: 9566–9573 16230422 10.1158/0008-5472.CAN-05-1003 PMC1403288

[bib45] Zhu Y, Spitz MR, Zheng YL, Hong WK, Wu XF (2002) BPDE-induced lymphocytic 3p21.3 aberrations may predict head and neck carcinoma risk. Cancer 95: 563–568 12209748 10.1002/cncr.10689

